# Therapeutic potential of *Atractylodes lancea* in restoring cardio-renal function in rats with diet-induced metabolic syndrome

**DOI:** 10.1186/s12906-025-05074-8

**Published:** 2025-09-30

**Authors:** Yun Jeong Yang, Mi Hyeon Hong, Jung Joo Yoon, Ai Lin Tai, Ho Sub Lee, Sung Joo Park, Hye Yoom Kim, Dae Gill Kang

**Affiliations:** 1 Hanbang Cardio-renal Research Center and Professional Graduate School of Oriental Medicine, Wonkwang University, Iksan, 54538 Korea; 2 College of Oriental Medicine and Professional Graduate School of Oriental Medicine, Wonkwang University, Iksan, 54538 Korea

**Keywords:** *Atractylodes lancea*, Metabolic syndrome, Insulin resistance, Cardiac function, Cardiac fibrosis, Cardiovascular

## Abstract

**Background:**

Cardio-Renal Metabolic Syndrome (CRS) encompasses metabolic disorders such as type 2 diabetes, hypertension, hyperlipidemia, chronic kidney disease, and heart failure. It is associated with obesity, systemic inflammation, and insulin resistance. *Atractylodes lancea* (AL), a traditional herbal remedy, has been previously reported to exhibit diuretic, sedative, antibacterial, and anticancer effects. However, the impact of AL on cardiovascular and renal functions within a metabolic syndrome (MS) model remains to be explored.

**Methods:**

Metabolic syndrome was induced in rats through an 8-week high-fat, high-fructose diet. After induction, experimental groups were orally administered olmesartan (10 mg/kg/day) or Aqueous extract of *Atractylodes lancea* (AAL) at 100 or 200 mg/kg/day for an additional 8 weeks. Body weight, fasting blood glucose, triglycerides, abdominal circumference, systolic blood pressure, and HDL-cholesterol levels were measured. Insulin levels and oral glucose tolerance tests (OGTT) were conducted to evaluate insulin resistance. Cardiac function was assessed using echocardiography, and ejection fraction and fractional shortening were analyzed. Masson’s trichrome and Picrosirius red staining were performed to evaluate fibrosis in the heart and aorta. Renal function was measured through creatinine clearance, blood urea nitrogen (BUN), and electrolyte levels. Periodic acid-Schiff (PAS) staining was additionally performed to evaluate histological changes in the kidney.

**Results:**

Administration of AAL resulted in significant reductions in body weight, fasting blood glucose, triglycerides, abdominal circumference, and systolic blood pressure, while HDL-cholesterol levels increased. AAL improved insulin resistance, as indicated by enhanced insulin levels and OGTT results. Echocardiography revealed improvements in ejection fraction and fractional shortening in AAL-treated groups compared to the MS group. Histological analysis showed that AAL reduced heart and aorta fibrosis, as well as attenuated kidney injury. Additionally, AAL improved renal function by enhancing creatinine clearance, reducing BUN levels, and stabilizing electrolyte balance.

**Conclusions:**

The aqueous extract of *Atractylodes lancea* (AAL) effectively ameliorated cardiovascular and renal dysfunction in a rat model of metabolic syndrome model. These results suggest that AAL may have preventive and therapeutic potential for cardio-renal complications associated with MS. However, further investigation is needed to evaluate its suitability for diet-based interventions and evaluate its safety and pharmacological profile.

**Supplementary Information:**

The online version contains supplementary material available at 10.1186/s12906-025-05074-8.

## Background

Metabolic syndrome (MS) is a complex and growing global health concern characterized by insulin resistance (IR), abdominal obesity, arterial hypertension, hyperglycemia, and dyslipidemia [1–3]. This syndrome presents a cluster of metabolic disorders that increase susceptibility to life-threatening diseases, including cardiovascular disease (CVD) and type 2 diabetes (T2DM). MS is primarily defined by reduced high-density lipoprotein cholesterol (HDL-C) levels, alongside elevated triglyceride and total cholesterol (T-Cho) levels [4, 5], making it a significant risk factor for coronary artery disease and progressive diabetic complications.

The rapid adoption of Westernized dietary patterns—characterized by high intakes of refined sugars, saturated fats, and processed foods—has significantly driven the increasing global prevalence of MS [6, 7]. Chronic consumption of high-fat, high-sugar diets promotes obesity, IR, and dyslipidemia, the core features of MS, while also inducing systemic inflammation that exacerbates metabolic dysfunction [8]. Given the growing prevalence of MS, new effective therapeutic strategies are urgently needed to prevent its progression to CVD, T2DM, and related complications.

The pathogenesis of MS involves several interconnected mechanisms, with IR playing a central role. IR impairs glucose metabolism, resulting in hyperglycemia, hyperinsulinemia, and eventually leads to β-cell dysfunction [9, 10]. It also contributes to vascular abnormalities, such as endothelial dysfunction and sympathetic nervous system hyperactivity, which promote hypertension and metabolic dysregulation [11]. These factors collectively accelerate CVD progression by promoting arterial stiffness, lipid accumulation, and systemic inflammation [12]. Long-term IR-related metabolic stress can further lead to myocardial remodeling, interstitial fibrosis, and cardiac dysfunction, culminating in heart failure [13].

MS also significantly impacts renal health. Visceral obesity, a hallmark of MS, induce renal inflammation, glomerular hyperfiltration, and glomerulosclerosis, thereby promoting the development of chronic kidney disease (CKD) [14]. Moreover, MS increases susceptibility to acute kidney injury (AKI), often exacerbated by hypertension and hyperlipidemia [15]. The nephropathy associated with MS reflects the interplay between metabolic, inflammatory, and hemodynamic stressors that progressively impair kidney function [16, 17].

Mechanistically, hyperglycemia and IR contribute to the disruption of the cardio-renal axis through multiple molecular pathways [18]. Excess intracellular glucose enhances mitochondrial reactive oxygen species (ROS) production, triggering oxidative damage and activating downstream signaling cascades such as the polyol pathway, protein kinase C, and the formation of advanced glycation end products (AGEs) [19, 20]. AGEs, through interacion with their receptor RAGE, promote inflammation, fibrosis, and increased tissue stiffness in both cardiac and renal tissues [21, 22]. In addition, dysregulation of nutrient-sensing pathways such as mTOR, and activation of the local renin-angiotensin-aldosterone system (RAAS), aggravate structural remodeling and organ dysfunction [23, 24]. Lipotoxicity resulting from elevated free fatty acid accumulation, and impaired mobilization of hematopoietic stem/progenitor cells (HSPCs), also contribute to cardiac and renal injury [25, 26]. These interconnected mechanisms collectively explain how metabolic disturbances drive progressive damage across the cardio-renal axis.

*Atractylodes lancea* Thunb (AAL), commonly referred to as Changchul (Cangzhu in Chinese), is a perennial herb used extensively in Korean and Oriental medicine for its therapeutic properties [27, 28]. Modern pharmacological studies have begun to elucidate the mechanisms underlying its bioactivity, demonstrating its potential to influence metabolic and inflammatory pathways. Recent in vitro studies have shown that sesquiterpenoids, such as atractylone and β-selinene, can modulate adipogenesis and lipid metabolism by activating AMPK (AMP-activated protein kinase), a critical regulator of energy homeostasis [29, 30]. Furthermore, atractylone has demonstrated anti-inflammatory effects by inhibiting NF-κB signaling and reducing pro-inflammatory cytokine expression in macrophages [31].

In vivo studies further support the potential of AAL in mitigating metabolic disorders. *Atractylodes lancea* extract has been reported to attenuate obesity and improve glucose tolerance in high-fat diet-induced obese mice, likely through modulation of PPAR-γ (peroxisome proliferator-activated receptor gamma) activity, which plays a key role in insulin sensitivity and lipid metabolism [32, 33]. Moreover, atractylone has demonstrated hepatoprotective effects by reducing oxidative stress and lipid accumulation in a non-alcoholic fatty liver disease (NAFLD) mouse model [34]. These findings highlight AAL’s potential to address key features of MS, including IR, obesity, and dyslipidemia.

Cardiovascular benefits of AAL have also been observed in animal studies. AAL extract was shown to reduce blood pressure in spontaneously hypertensive rats (SHRs) by enhancing endothelial nitric oxide (NO) production and improving vascular function [35]. Additionally, sesquiterpenoids in AAL have exhibited cardioprotective effects by attenuating myocardial oxidative damage and inflammation in ischemia-reperfusion injury models [36, 37]. These results suggest that AAL may alleviate cardiovascular complications commonly associated with MS.

Renal protective effects of AAL have been documented as well. In a study using a rat model of renal fibrosis, atractylone reduced renal inflammation and fibrosis by modulating TGF-β/Smad signaling, a pathway closely linked to chronic kidney disease progression [38]. Furthermore, AAL extract has demonstrated diuretic and anti-inflammatory effects in animal models of acute kidney injury, supporting its relevance in managing nephropathy associated with MS [39, 40].

Despite its extensive traditional use, the effects of AAL on MS, particularly in models induced by high-fat and high-fructose (HFHFr) diets, remain underexplored. HF and HFr diets are widely used to replicate the pathophysiological features of human MS in experimental models, promoting obesity, IR, hyperlipidemia, and systemic inflammation [41, 42]. Given the preclinical evidence supporting the anti-inflammatory, anti-obesity, and metabolic regulatory effects of AAL, investigating its potential to mitigate cardio-renal dysfunction caused by prolonged MS is warranted. This study aims to address this gap by evaluating the therapeutic effects of the aqueous extract of *Atractylodes lancea* (AAL) on cardio-renal dysfunction induced by long-term MS. By assessing multiple metabolic parameters and histological markers, this research seeks to elucidate the effects of AAL on cardiovascular and renal functions. The findings of this study may contribute to the development of herbal strategies or nutraceutical approaches for managing MS and alleviating the burden of its associated complications.

## Methods

### Preparation of *Atractylodes lancea* (AAL)

AAL was purchased from the Herbal Medicine Co-operative Association, Iksan, Jeonbuk Province, Korea, in September 2021. The dried AAL (500 g) was boiled with 2500 mL of distilled water (DW) for 2 h using a vacuum evaporator (N-11, Tokyo Rikakikai, Tokyo, Japan). After boiling, the extract was concentrated and dried (yield: 358.8 g, 76%) and stored at −70 °C until use.

### HPLC analysis of atractylodin in AAL extracts

High-performance liquid chromatography (HPLC) was performed to confirm the presence of atractylodin in the AAL extracts. The analysis was conducted using a Capcell pak UG80 column (4.6 mm I.D. x 150 mm, 5 μm, Osaka Soda, Japan) at a column temperature of 40 ℃. The mobile phase consisted of purified water (330 mL) containing phosphoric acid (0.6 mL) mixed with acetonitrile (670 mL), at a flow rate of 1.0 mL/min for a 30-minute run. Detection was carried out at a wavelength of 340 nm. A 10 µL aliquot of each sample, including the standard (STD) solution of atractylodin and the AAL extracts (samples 1, 2, and 3), was injected into the HPLC system. Peaks corresponding to atractylodin were identified based on retention time by comparison with the standard solution. The consistency of the atractylodin peak across samples validated the extraction process, confirming the reliability and reproducibility of the herbal preparations.

### Animal model and diet

Animal model and diet All experimental procedures were conducted in accordance with the National Institute of Health Guide for the Care and Use of Laboratory Animals and approved by the Institutional Animal Care and Utilization Committee for Medical Science of Wonkwang University (WKU 22–52). The study used nine-week-old male Wistar rats (Samtako, Osan, Korea), which were acclimatized for one week before being randomly assigned to five groups, with seven rats per group; Control group (Cont), Negative control group (HFHFr), Positive control group (OMT), AAL low-dose treatment group (AAL1), and an AAL high-dose treatment group (AAL2). The Cont received a standard chow diet (10% kcal fat, D12450, Research Diets, Inc., New Brunswick, NJ, USA) with DW, while the HFHFr, OMT, AAL1, AAL2 groups were fed a high-fat diet (45% kcal fat, D12451, Research Diets, Inc.) with 10% fructose water (Daejung, Siheung, Korea). All diets were provide for 16 weeks. Diet composition is detailed in Table 1. Animals were housed under controlled conditions with a constant temperature, relative humidity, and a 12-hour light/dark cycle. After eight weeks on the respective diets, AAL extract and olmesartan were administered orally through oral gavage needle for an additional eight weeks, while the Cont and HFHFr groups received DW. OMT, an angiotensin receptor blocker (ARB), served as a positive control for its known blood pressure-lowering effects through vascular relaxation [43]. Body weight (BW) was recorded weekly throughout the experiment.

**Table 1:** Composition of high fat diet (g)

Ingredients		Experimental diets^1)^	
		Normal Diet	High Fat Diet
Casein, Lactic, 30 Mesh		200.0 g	200.0 g
Cystine, L		3.0 g	3.0 g
Starch, Corn		452.2 g	176.8 g
Sucrose, Fine Granulated		176.8 g	100.0 g
Maltodextrin (Lodex 10)		75.0 g	72.8 g
Cellulose (Solka Floc, FCC200)		50.02 g	50.02 g
Soybean Oil, USP		25.0 g	177.5 g
Lard		20.0 g	25.0 g
Mineral mix^2)^		50.02 g	50.02 g
Choline Bitartrate		2.0 g	2.0 g
Vitamin mix^3)^		1.0 g	1.0 g
Calorie (kcal%)	Carbohydrate	70%	35%
	Protein	20%	20%
	Fat	10%	45%

^(1)^ Normal diet containing 10 kcal% fat (D12450H, Research Diets. Inc., NJ, USA); high fat diet containing 45 kcal% fat (D12451, Research Diets. Inc., NJ, USA)

^(2)^ Mineral mix: Mineral mix S10026B (Research Diets. Inc., NJ, USA)

^(3)^ Vitamin mix: Vitamin mix V10001C (Research Diets. Inc., NJ, USA)

### Estimation of oral glucose tolerance test (OGTT)

The oral glucose tolerance test (OGTT) was conducted prior to sacrifice. After 15 h of overnight fasting, blood samples were collected from the tail vein using a glucometer (OneTouch^®^ Ultra™) and test strips (i-SENS, Inc., Seoul, Korea) to measure baseline glucose levels. A glucose solution (2 g/kg body weight) was then administered orally via gavage. Additional blood samples were collected from the tail vein at 30, 60, 90, and 120 min following glucose administration to monitor blood glucose levels.

### Euthanasia and tissue collection

At the end of the study, animals were euthanized in accordance with ethical guidelines. Euthanasia was performed using isoflurane anesthesia to ensure the animals were unconscious before sacrifice. Isoflurane was administered using the Harvard Apparatus Animal Isoflurane System, specifically designed for small animal procedures (Harvard Apparatus, Holliston, MA, USA). The anesthesia induction was set to a concentration of 4% for induction and maintained at 2% during tissue collection. This method was chosen to minimize pain and distress in compliance with ethical standards. Blood was collected via cardiac puncture while under anesthesia, plasma separated for analysis, and tissues were harvested and stored at −70 °C for further biochemical investigations.

### Analysis of plasma biochemicals

The levels of glucose, triglyceride (TG), T-Cho, HDL-C, creatine phosphokinase (CPK), creatine kinase myocardial band (CK-MB), lactate dehydrogenase (LDH), creatinine (Cre), and blood urea nitrogen (BUN) in plasma were measured using an automated clinical chemistry analyzer (FUJI DRI-CHEM NX700, FUJIFILM Corporation, Tokyo, Japan). Plasma insulin levels were determined using a commercial enzyme-linked immunosorbent assay (ELISA) kit (FUJIFILM Wako Shibayagi, Virginia, USA), and leptin levels were measured using an ELISA kit from Abcam (Cambridge, UK). The homeostasis model assessment of insulin resistance (HOMA-IR) was calculated according to Matthews et al. [44] $$\mathrm{HOMA}-\mathrm{IR}\;=$$$$\left(\mathrm{fasting}\;\mathrm{plasma}\;\mathrm{insulin}\;\right.$$$$\left.\times\mathrm{fasting}\;\mathrm{blood}\;\mathrm{glucose}\right)/405$$. The Cardiac risk ratio was calculated according to Kinosian et al. [45] using this formula : $$\mathrm{Cardiac}\;\mathrm{risk}\;\mathrm{ratio}\;=\mathrm T-\mathrm{Cho}\;/\mathrm{HDL}-\mathrm C$$.

### Assessment of cardiac function via echocardiography

Echocardiography was performed using an ultrasound unit with an 18LS probe set at a frequency of 14 MHz (VINNO6, Vinno Corporation, China). Animals were anesthetized with 4% isoflurane mixed with oxygen, administered via a nose cone while placed on a thermal mat to maintain body temperature. During measurements, isoflurane levels were reduced to 1.5% at a flow rate of 1 L/min to sustain anesthesia. After 16 weeks of the experiment, echocardiographic measurements were taken for each rat. Ejection fraction (EF) was determined using VINNO LV function software, and fractional shortening (FS) was calculated based on the left ventricular intraventricular diameters at systole and diastole (LVIDs and LVIDd, respectively).

### Assessment of electrocardiography (ECG)

Electrocardiography was recorded continuously using standard three-lead skin electrodes. Two electrodes were placed on the right and left forelimbs, with a neutral electrode on the hind limb. The ECG data was collected using electrodes connected to the data acquisition system, PowerLab (AD Instruments, Australia).

### Measurement of systolic blood pressure (SBP)

Systolic blood pressure was measured using the non-invasive tail-cuff plethysmography method. An automatic sphygmomanometer (CODA^®^, Torrington, USA) was used to record the measurements.

### Preparation of aortic rings and recording of vascular relaxation

The thoracic aorta was rapidly excised from the rats and cleaned of fat tissue in Krebs’ solution (118.0 mM NaCl, 1.1 MgSO_4_, 4.7 KCl, 1.2 KH_2_PO_4_, 25.0 NaHCO_3_, 10.0 glucose, and 1.5 CaCl_2_, pH 7.4). The aorta was sectioned into 3 mm rings and suspended in an organ chamber containing 5 mL of Krebs solution. After pre-treatment with phenylephrine (PE) for 5 min, acetylcholine (ACh) and sodium nitroprusside (SNP) were added at specific concentrations to assess vascular relaxation.

### Monitoring of renal function

Animals were housed in individual metabolic cages for 24 h to collect urine and monitor food and water intake. From day 15 at 9:00 a.m. to day 16 at 9:00 a.m., 24-hour urine samples were collected to analyze Cr), sodium, chloride, potassium, and urinary osmolality (Uosmol). Ion concentrations were measured using an electrolyte analyzer (NOVA 4, Biochemical, Waltham, MA, USA), and Uosmol was determined with an Advanced CRYOMATIC™ osmometer (Model 3900, Advanced Instruments Inc., Norwood, MS, USA). Urine creatinine was measured using a colorimetric method with a spectrophotometer (Milton Roy, Rochester, NY, USA). Creatinine clearance (Ccr) was calculated using the following formula: $$\mathrm{Ccr}\left(\mathrm{mL}/\min/\mathrm{kg}\right)=\mathrm{urine}\;\mathrm{Cre}\;\left(\mathrm{mg}/\mathrm{mL}\right)\times\mathrm{urinary}\;$$$$\mathrm{volume}\;\left(\mathrm{UV},\;\mathrm{mL}/\mathrm{kg}/\min\right)/\mathrm{plasma}\;\mathrm{Cre}\;\left(\mathrm{mg}/\mathrm{mL}\right)$$.

### Histological analysis

Epididymal fat, heart, vascular, and kidney tissues were collected from each rat and fixed in 10% formalin prepared in 0.01 M phosphate-buffered saline for seven days. The tissues were embedded in Optimal Cutting Temperature (OCT) compound and paraffin, sectioned at 6–8 μm, and mounted on slides. Epididymal fat sections were stained with Oil Red O for histopathological evaluation. Heart and vascular tissues were stained using Masson’s trichrome staining (Masson’s Trichrome Stain Kit, BBC Biochemical, Mount Vernon, WA, USA) to assess fibrosis. Heart tissue was further stained with Picrosirius Red (Polysciences, Inc., Warrington, PA, USA). Morphological changes in kidney tissues were analyzed using periodic acid-Schiff (PAS) staining. PAS-stained slides were analyzed using Motic Easy Scan Pro 1 (microscope slide scanner, National Optical & Scientific Instruments, Inc., Schertz, TX, USA). The remaning imaging analyses were conducted by EVOS™ M5000 (Thermo Fisher Scientific, Bothell, WA, USA). ImageJ software (NIH, USA) was used to quantify glomerular surface area from PAS-stained kidnyey sections and fibrosis percentage from Masson’s trichrome-stained cardiac and vascular tissues using a standardized region of interest (ROI). All analyses were performed in a blinded manner.

### Statistical analysis

All experiments were independently performed at last three times and statistical analysis was conducted using Sigma 10.0 software, and results were expressed as mean ± standard error (S.E.). Differences between group means were evaluated using Student’s t-test, and a *P* value < 0.05 was considered statistically significant.

## Results

### Detection and stability of atractylodin in extracts of *Atractylodes lancea* via HPLC analysis

**Fig. 1:**
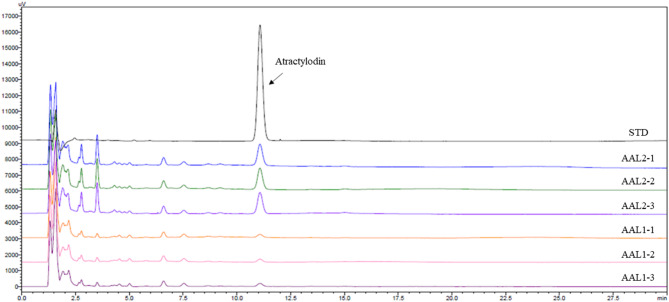
HPLC Chromatogram of Atractylodin. The chromatogram displays the high-performance liquid chromatography (HPLC) analysis of atractylodin, a bioactive compound in Atractylodes lancea. The standard sample chromatogram (STD) shows a distinct peak corresponding to atractylodin, indicated by the black arrow

The high-performance liquid chromatography (HPLC) analysis confirmed the presence of atractylodin, a key bioactive compound, in AAL extracts (Fig. 1). As indicated by the sharp peak in the standard (STD) chromatogram, the same peak was consistently detected across multiple extract samples (AAL 1, 2, and 3), confirming the presence of atractylodin. The peak intensity was comparable to the standard, indicating efficient extraction and stability of atractylodin in the samples. The reproducibility of the atractylodin peaks across different batches demonstrates the reliability of the extraction process, ensuring the consistent quality of *Atractylodes lancea* formulations for further experimental applications.

### Effects of AAL on abdominal adiposity and lipid metabolism in rats with high-fat-high-fructose diet-induced metabolic syndrome

**Fig. 2:**
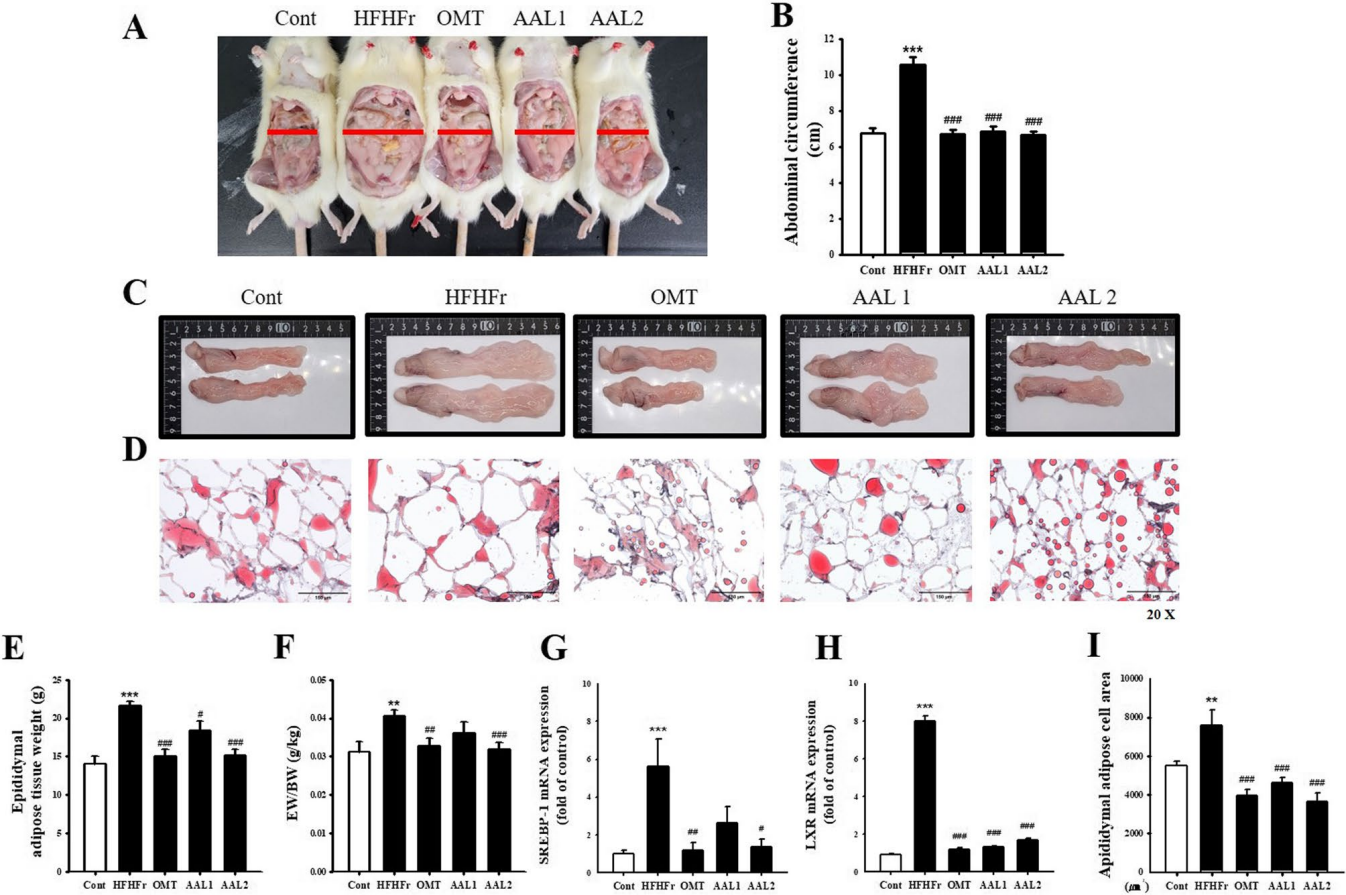
Effects of *Atractylodes lancea* on Adipose Tissue and Lipid Metabolism in HFHFr Diet-Induced Metabolic Syndrome Rats. **A** Representative images of abdominal fat in rats after 16 weeks, showing enlarged visceral fat in the HFHFr group compared to the control, with reductions in AAL (100 mg/kg and 200 mg/kg) and olmesartan (OMT) treated groups. **B** Abdominal circumference measurements, showing a significant reduction in the AAL-treated groups. **C** Adipose tissue images highlighting reduced fat accumulation with AAL treatment. **D** Histological sections of adipose tissue stained with H&E, revealing smaller adipocytes in AAL-treated groups. **E** Weight of epididymal adipose tissue, showing a significant reduction with AAL. **F** EW/BW ratio was significantly reduced by AAL. **G**, **H** SREBP-1c and LXR mRNA levels were downregulated with AAL treatment, indicating improved lipid metabolism. **I** Mean adipocyte cell area was reduced with AAL treatment, highlighting its anti-obesity potential. *P < 0.05, **P < 0.01, ***P < 0.001 vs. control; #*P* < 0.05, ##*P* < 0.01, ###*P* < 0.001 vs. HFHFr group

The HFHFr group exhibited a significant increase in abdominal circumference compared to the control group, while treatment with OMT and AAL (both doses) significantly reduced this increase (Fig. 2A and B). Morphological analysis of epididymal fat (Fig. 2C and D) revealed that HFHFr-induced rats developed enlarged and disorganized adipocytes, whereas AAL treatment improved adipocyte structure and reduced cell size, comparable to OMT treatment. The weight of epididymal adipose tissue (Fig. 2E) and the fat-to-body weight ratio (EW/BW; Fig. 2F) were significantly elevated in the HFHFr group, and both OMT and AAL treatments reduced these parameters, with AAL2 showing the most pronounced effect. Gene expression analysis (Fig. 2G and H) indicated that key Lipid metabolism regulators, sterol regulatory element-binding protein 1c (SREBP-1c) and liver X receptor (LXR), were significantly upregulated in the HFHFr group, whereas both AAL doses markedly suppressed their expression. Furthermore, the HFHFr group exhibited significantly enlarged adipocyte cell areas (Fig. 2I), which were reduced by AAL and OMT treatment.

### Effects of AAL on glucose homeostasis, insulin sensitivity, and adipokine regulation in metabolic syndrome rats

**Fig. 3:**
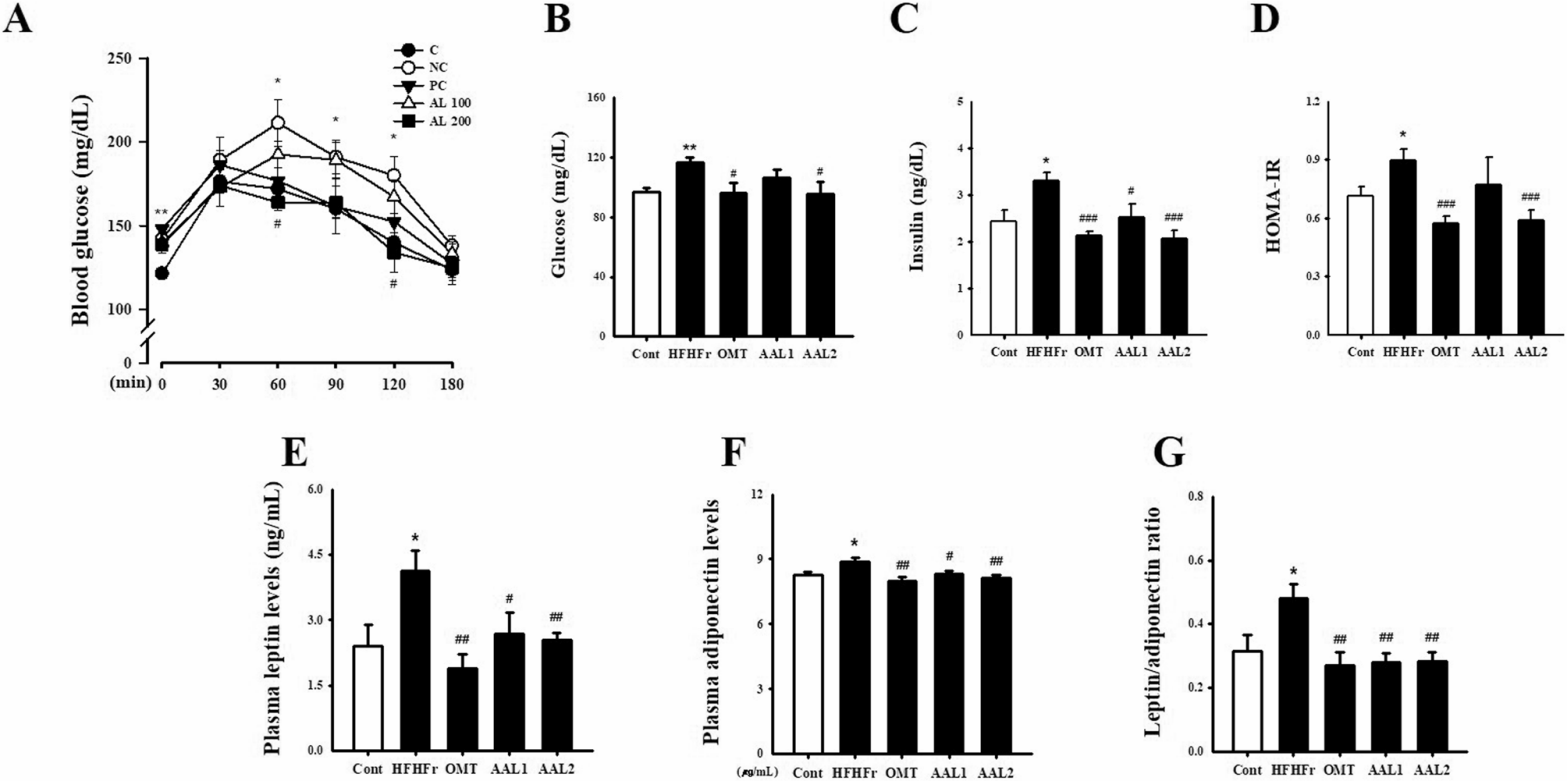
Effects of *Atractylodes lancea* on Glucose Homeostasis and Adipokine Levels in HFHFr Diet-Induced Metabolic Syndrome Rats. **A** Fasting blood glucose levels, showing significant reductions with AAL treatment. **B** AUC from the OGTT, indicating improved glucose tolerance in AAL-treated groups. **C** Plasma insulin levels, showing reductions with AAL. **D** HOMA-IR scores, with significant decreases in insulin resistance in AAL-treated groups. **E**-**G** Plasma leptin levels were lowered, while adiponectin levels increased with AAL treatment, reflecting improved metabolic regulation. **H** The leptin/adiponectin ratio was normalized with AAL. *P < 0.05, **P < 0.01, ***P < 0.001 vs. control; #*P* < 0.05, ##*P* < 0.01, ###*P* < 0.001 vs. HFHFr group

The OGTT revealed significantly elevated blood glucose levels in the HFHFr group at various time points compared to the control group, indicating impaired glucose tolerance (Fig. 3A). Treatment with AAL (100 and 200 mg/kg) and OMT notably reduced glucose levels, particularly at the 60 and 120-minute time points. The fasting blood glucose levels (Fig. 3B) were significantly increased in the HFHFr group (*p* < 0.01) compared to the control, whereas AAL and OMT treatments reduced glucose levels (*p* < 0.05). Insulin levels (Fig. 3C) and HOMA-IR scores (Fig. 3D) were significantly elevated in the HFHFr group (*p* < 0.05), indicating insulin resistance, while both doses of AAL and OMT significantly lowered these markers (*p* < 0.001). Plasma leptin levels (Fig. 3E) were higher in the HFHFr group compared to the control group, and AAL treatment effectively reduced leptin concentrations, comparable to the OMT group. Conversely, plasma adiponectin levels (Fig. 3F) were significantly increased in the HFHFr group, but AAL and OMT treatments restored these levels toward normal (*p* < 0.01). The leptin/adiponectin ratio (Fig. 3G) was markedly increased in the HFHFr group (*p* < 0.05) and significantly reduced by AAL and OMT treatment (*p* < 0.01).

### Effects of AAL on serum lipid profiles in metabolic syndrome rats

**Fig. 4:**
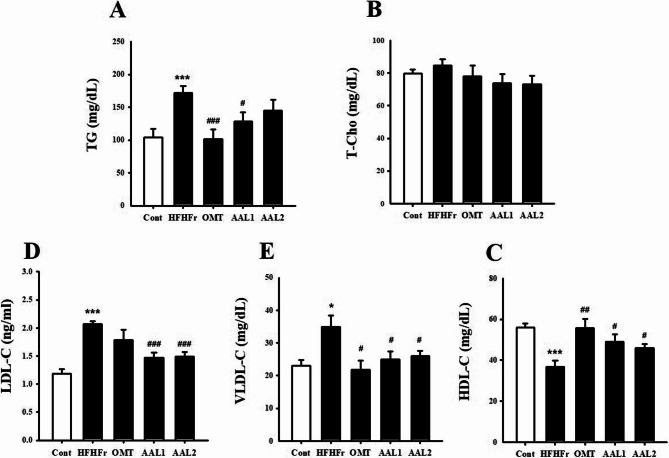
Effects of *Atractylodes lancea* on Lipid Profiles in HFHFr Diet-Induced Metabolic Syndrome Rats. **A** Triglycerides, (**C**) LDL-C, and (**D**) VLDL-C levels were significantly reduced, while (**E**) HDL-C levels were restored with AAL treatment, demonstrating beneficial effects on lipid profiles. *P < 0.05, **P < 0.01, ***P < 0.001 vs. control; #*P* < 0.05, ##*P* < 0.01, ###*P* < 0.001 vs. HFHFr group

In Fig. 4A, TG levels were significantly elevated in the HFHFr group compared to the control group (*p* < 0.001). Treatment with AAL 200 mg/kg treatment reduced TG levels, while treatment OMT and AAL 100 mg/kg significantly decreased compared to the HFHFr group. However, T-Cho levels (Fig. 4B) did not differ significantly across the groups. Low-density lipoprotein cholesterol (LDL-C) levels (Fig. 4D) were markedly increased in the HFHFr group (*p* < 0.001), and both AAL doses effectively lowered LDL-C levels (*p* < 0.001) to values comparable to the OMT group. Very low-density lipoprotein cholesterol (VLDL-C) levels (Fig. 4E) were also elevated in the HFHFr group (*p* < 0.05), while AAL and OMT treatments significantly reduced these levels (*p* < 0.05). Conversely, high-density lipoprotein cholesterol (HDL-C) levels (Fig. 4C) were significantly reduced in the HFHFr group (*p* < 0.001) compared to the control, but both AAL doses and OMT treatment restored HDL-C levels (*p* < 0.01).

### Effects of AAL on cardiac function and structure in metabolic syndrome rats

**Fig. 5:**
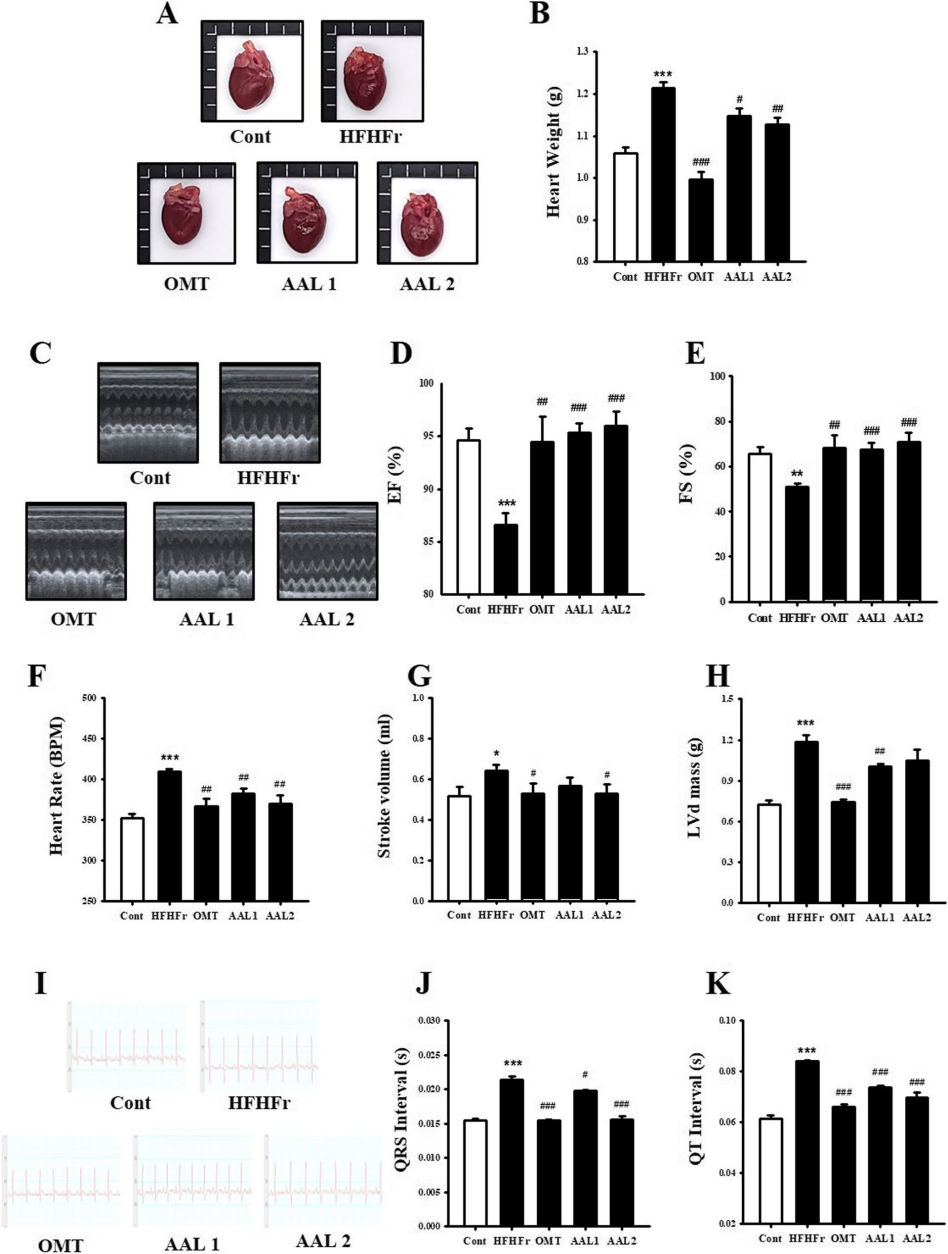
Effects of *Atractylodes lancea* on Cardiac Structure and Function in HFHFr Diet-Induced Metabolic Syndrome Rats. **A** Heart images showing reduced size in AAL-treated groups. **B** Heart weight measurements, with reductions in AAL groups. **C**-**E** Echocardiographic findings, showing improved ejection fraction (EF) and fractional shortening (FS) with AAL treatment. **F**-**H** Heart rate, stroke volume, and left ventricular mass (LVd mass) were normalized by AAL. *P < 0.05, **P < 0.01, ***P < 0.001 vs. control; #*P* < 0.05, ##*P* < 0.01, ###*P* < 0.001 vs. HFHFr group

The HFHFr group exhibited visibly enlarged hearts compared to the control group, while treatment with OMT and AAL (both doses) reduced heart size (Fig. 5A). Figure 5B shows that heart weight was significantly higher in the HFHFr group (*p* < 0.001) compared to the control, but both AAL and OMT treatments significantly decreased heart weight (*p* < 0.05, *p* < 0.01). Echocardiographic images (Fig. 5C) revealed impaired cardiac function in the HFHFr group, confirmed by the reduction in ejection fraction (EF, Fig. 5D) and fractional shortening (FS, Fig. 5E). Treatment with AAL significantly improved both EF and FS compared to the HFHFr group (*p* < 0.01). Heart rate (BPM) analysis (Fig. 5F) indicated a significant increase in the HFHFr group (*p* < 0.001), which was normalized following AAL and OMT treatments (*p* < 0.01). Stroke volume (Fig. 5G) was significantly increased in the HFHFr group, but AAL treatment restored it toward normal levels (*p* < 0.05). The left ventricular mass (LVd mass, Fig. 5H) was markedly increased in the HFHFr group (*p* < 0.001), while AAL and OMT treatments significantly reduced LVd mass (*p* < 0.01). Electrocardiographic (ECG) recordings (Fig. 5I) showed abnormal QRS and QT intervals in the HFHFr group, indicating impaired electrical activity. As shown in Fig. 5J, the QRS interval was significantly prolonged in the HFHFr group (*p* < 0.001), while AAL and OMT treatments shortened the QRS interval (*p* < 0.05, *p* < 0.01). Similarly, the QT interval (Fig. 5K) was significantly prolonged in the HFHFr group (*p* < 0.01), but treatment with AAL and OMT restored it to normal values (*p* < 0.01, *p* < 0.001). LVEDV and LVESV values were included in the supplementary data to support the interpretation (Supplementary data).

### Ameliorative effects of AAL on cardiac fibrosis, injury markers, and inflammatory responses in metabolic syndrome rats

**Fig. 6:**
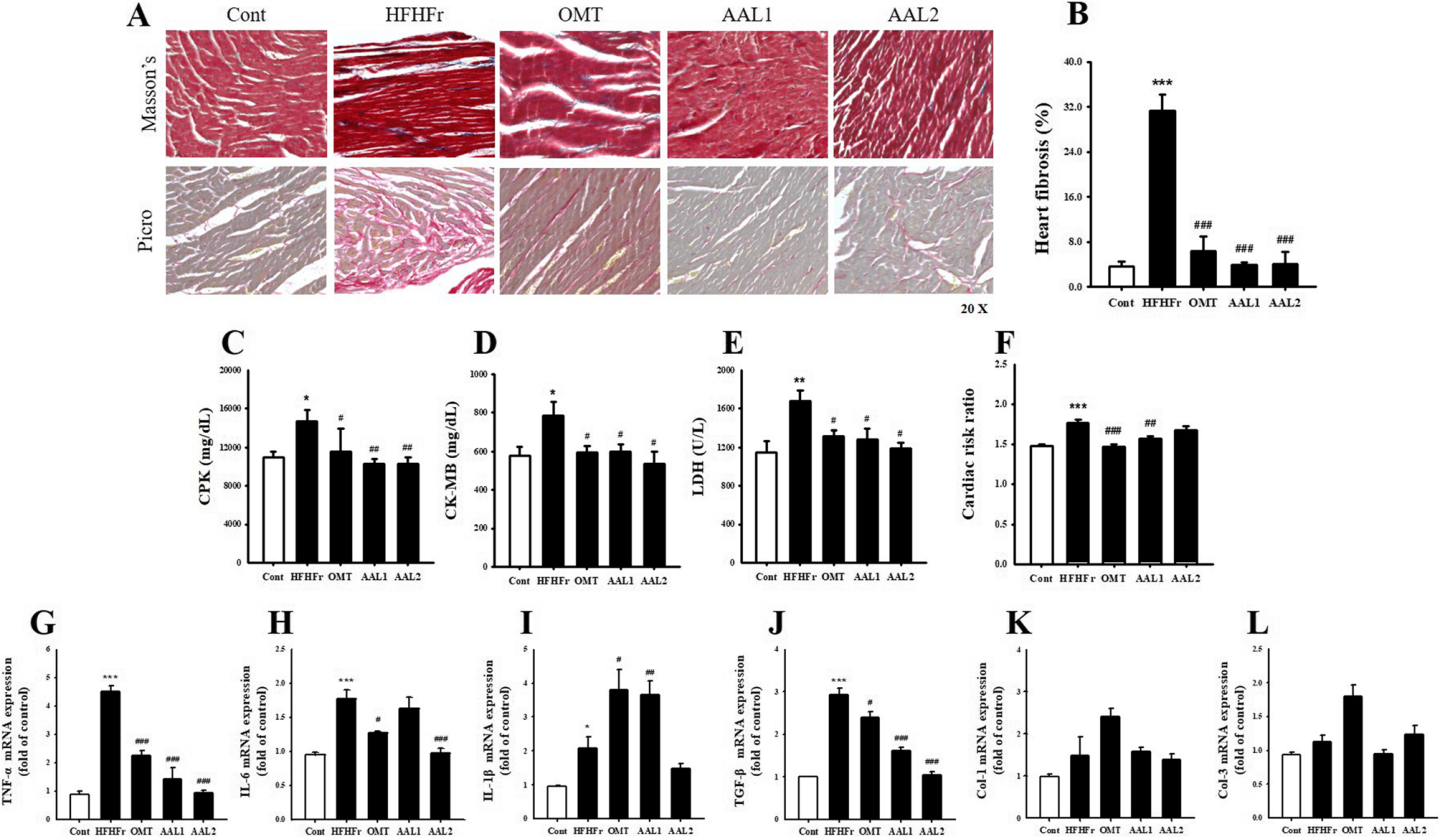
Effects of *Atractylodes lancea* on Cardiac Fibrosis, Injury Markers, and Inflammatory Responses in HFHFr Diet-Induced Metabolic Syndrome Rats. **A**-**B** Masson’s trichrome and Picrosirius red staining images showing reduced fibrosis with AAL treatment. **C**-**E** Plasma levels of CPK, CK-MB, and LDH, showing decreases with AAL. **F** Cardiac risk ratio improved with AAL treatment. **G**-**L** Pro-inflammatory and profibrotic markers were significantly reduced with AAL, indicating reduced fibrosis and inflammation. *P < 0.05, **P < 0.01, ***P < 0.001 vs. control; #*P* < 0.05, ##*P* < 0.01, ###*P* < 0.001 vs. HFHFr group

Figure 6A shows representative Masson’s trichrome and Picrosirius red staining images, where the HFHFr group exhibited extensive cardiac fibrosis, characterized by increased collagen deposition. In contrast, AAL treatment significantly improved cardiac structure, with reduced fibrosis comparable to the OMT group. Quantification of cardiac fibrosis in Fig. 6B reveals that the HFHFr group showed a marked increase in fibrosis percentage (*p* < 0.001) compared to the control group, while both doses of AAL (AAL1 and AAL2) significantly reduced fibrosis (*p* < 0.01, *p* < 0.001). Cardiac injury markers were also elevated in the HFHFr group, including CPK (Fig. 6C), CK-MB (Fig. 6D), and LDH (Fig. 6E), indicating myocardial damage. Both AAL and OMT treatments significantly reduced the levels of these biomarkers, restoring them toward normal values (*p* < 0.05, *p* < 0.01). Similarly, the cardiac risk ratio (Fig. 6F) was significantly higher in the HFHFr group (*p* < 0.001) and was lowered by AAL treatments (*p* < 0.01, *p* < 0.001). Gene expression analyses of inflammatory markers show that TNF-α (Fig. 6G) and interleukins (IL-6, Fig. 6H; IL-1β, Fig. 6I) were markedly upregulated in the HFHFr group (*p* < 0.001). AAL treatment significantly downregulated these pro-inflammatory cytokines. However, IL-1β tended to decrease but was not significant. Furthermore, the expression of profibrotic markers, TGF-β (Fig. 6J) was significantly increased in the HFHFr group (*p* < 0.05, *p* < 0.001). AAL treatments, especially at higher doses, significantly reduced the expression of these markers (*p* < 0.01), indicating an attenuation of cardiac fibrosis. Collagen isoforms Col1a1 and Col3a1 tended to decrease but was not significant (Fig. 6K and L).

### Effects of AAL on vascular function and structure in metabolic syndrome rats

**Fig. 7:**
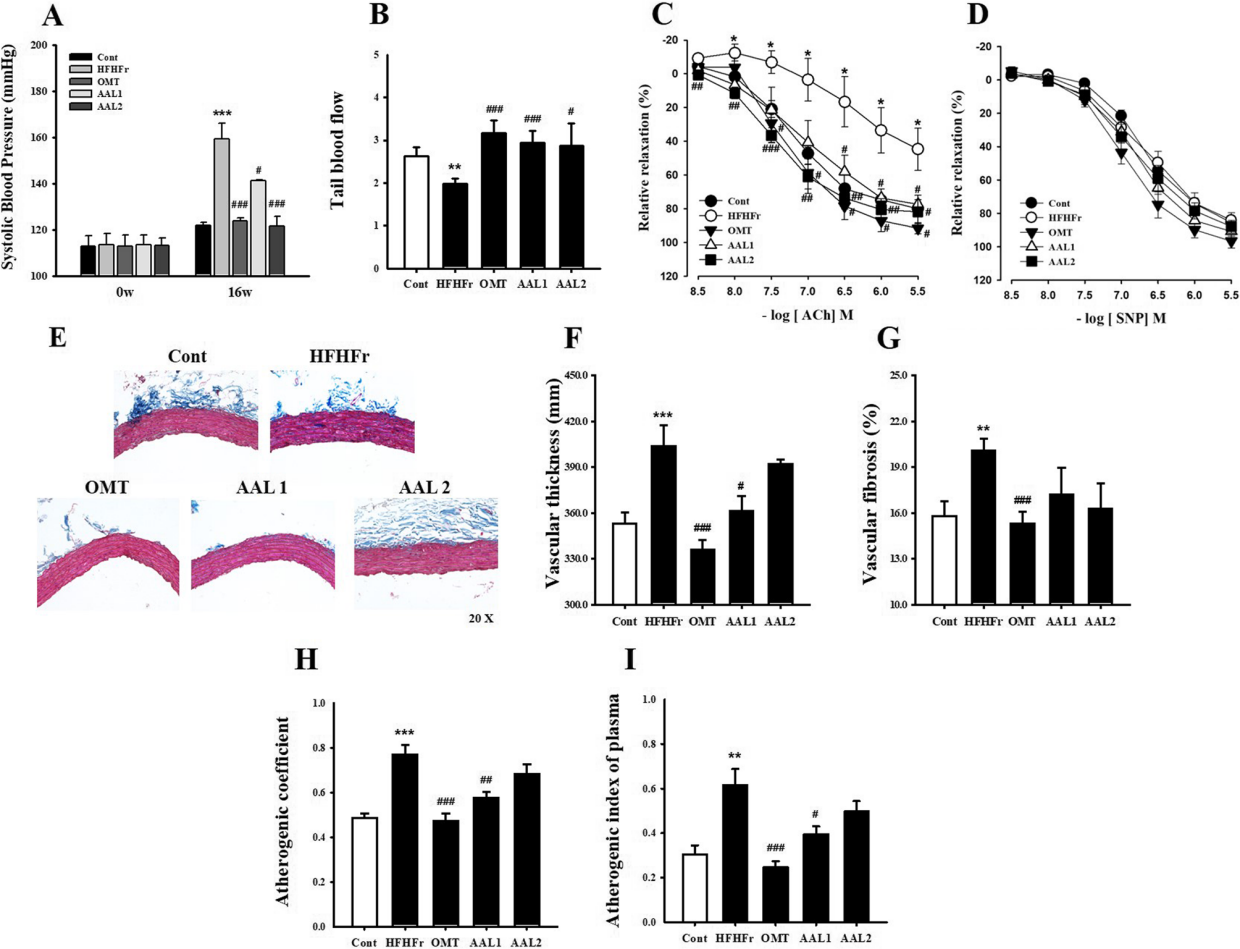
Effects of *Atractylodes lancea* on Vascular Function and Atherogenic Markers in HFHFr Diet-Induced Metabolic Syndrome Rats. **A** Systolic blood pressure significantly reduced with AAL treatment. **B** Tail blood flow restored in AAL-treated groups. **C**-**D** Enhanced vascular relaxation responses to ACh and SNP with AAL. **E**-**G** Reduced vascular fibrosis and thickness, along with improved atherogenic coefficient and index with AAL. *P < 0.05, **P < 0.01, ***P < 0.001 vs. control; #*P* < 0.05, ##*P* < 0.01, ###*P* < 0.001 vs. HFHFr group

Figure 7A shows that the HFHFr group exhibited a significant increase in systolic blood pressure after 16 weeks compared to the control group (*p* < 0.001), while both doses of AAL and OMT treatments significantly lowered blood pressure (*p* < 0.05, *p* < 0.001). Similarly, Fig. 7B indicates that tail blood flow, which was reduced in the HFHFr group (*p* < 0.01), was restored by AAL and OMT treatments (*p* < 0.05, *p* < 0.01). The vascular relaxation response to ACh and SNP is depicted in Fig. 7C and D, respectively. The HFHFr group exhibited impaired endothelium-dependent and endothelium-independent relaxation responses, while treatment with AAL and OMT significantly improved vascular relaxation, indicating enhanced vascular function (*p* < 0.05). Figure 7E shows histological images of vascular tissue stained for thickness and fibrosis. The HFHFr group exhibited marked vascular thickening and fibrosis compared to the control, whereas AAL and OMT treatments reduced these structural abnormalities. Quantification of vascular thickness (Fig. 7F) shows a significant increase in the HFHFr group (*p* < 0.001) compared to the control, with both AAL and OMT treatments reducing thickness (*p* < 0.05, *p* < 0.001). Similarly, vascular fibrosis (Fig. 7G) was significantly higher in the HFHFr group (*p* < 0.01) and was effectively reduced by OMT treatments (*p* < 0.001). AAL treatment reduced the vascular fibrosis, but there was no significant. Additionally, the HFHFr group displayed elevated atherogenic indices, including the atherogenic coefficient (Fig. 7H) and atherogenic index of plasma (Fig. 7I), which were significantly reduced by AAL and OMT treatments (*p* < 0.01, *p* < 0.001).

### Effects of AAL on renal function and structure in metabolic syndrome rats

**Fig. 8:**
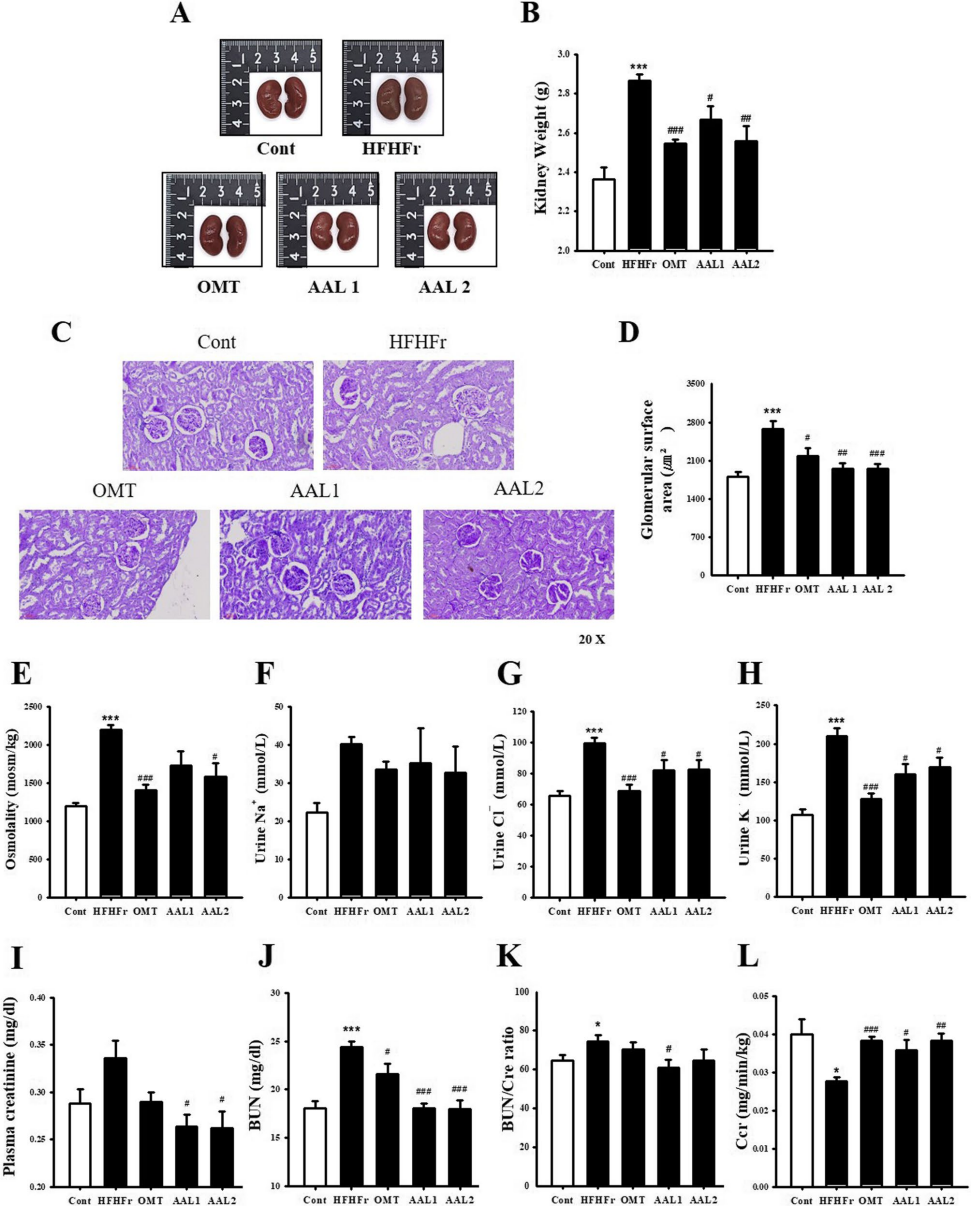
Effects of Atractylodes lancea on Renal Function and Structure in HFHFr Diet-Induced Metabolic Syndrome Rats. **A** Kidney images showing reduced size in AAL-treated groups. **B** Kidney weight measurements, with reductions in AAL groups. **C** PAS staining images showing restored renal morphology with AAL treatment. **D** glomerular surface area (µm²). **E**-**H** Urine levels of Osmolality, Na^+^, Cl^−^, K^+^ levels. **I**-**L** Renal function ratio improved with AAL treatment. *P < 0.05, **P < 0.01, ***P < 0.001 vs. control; #*P* < 0.05, ##*P* < 0.01, ###*P* < 0.001 vs. HFHFr group

The HFHFr group exhibited visibly enlarged kidneys compared to the control group, while treatment with OMT and AAL (both doses) reduced kidney size (Fig. 8A). Kidney weight (Fig. 8B) was significantly higher in the HFHFr group (*p* < 0.001) compared to the control, but both AL and OMT treatments significantly decreased kidney weight (*p* < 0.05, *p* < 0.01, *p* < 0.001). PAS staining (Fig. 8C) revealed morphological alterations in the HFHFr group, including glomerular hypertrophy (Fig. 8D) and narrowing of the capillary lumen in some glomeruli. Furthermore, excessive deposition of PAS-positive substances was observed in both the glomerular basement membrane (GBM) and tubular basement membrane (TBM), indicating basement membrane thickening. A slight expansion of the renal interstitial space was also noted. OMT treatment partially ameliorated HFHFr-induced renal injury, while AAL treatment effectively restored glomerular hypertrophy, capillary lumen narrowing, GBM and TBM alterations, basement membrane thickening, and interstitial expansion. In particular, AAL1 restored these parameters to levels comparable to those in the control group. Uosmol (Fig. 8E), urine Na^+^ (Fig. 8F), Cl^−^ (Fig. 8G), and K^+^ (Fig. 8H) were confirmed to measure the equilibrium state of the electrolyte in the body by using urine. Due to the HFHFr, all of the factors increased compared to the Cont group, and significantly decreased with OMT. Osmolality was effectively reduced by the treatment of AAL2, and Cl^−^ and K^+^ were significantly reduced in both AAL1 and AAL2 treatment groups, but no significant effect was observed for Na^+^ levels. Cre (Fig. 8I), BUN (Fig. 8J), BUN/Cre ratio (Fig. 8K), and Ccr (Fig. 8L) were measured to measure kidney function. Plasma Cre, plasma BUN, and BUN/Cre ratio increased compared to Cont due to the HFHFr diet, and Ccr decreased. Two volumes of AAL were reversed these levels.

## Discussion

This study highlights the therapeutic potential of AAL in improving metabolic, cardiovascular, and renal abnormalities in a rat model of HFHFr diet-induced MS. MS is increasingly recognized as a systemic disorder characterized by complex interactions between central metabolic disturbances and end-organ damage, particularly affecting the heart and kidneys [46]. Although therapeutic strategies have largely focused on glycemic and lipid control, treatment options that improve both cardiovascular and renal outcomes remain limited. In this context, the findings of this study suggest that AAL exerts multi-organ benefits by modulating insulin resistance, systemic inflammation, and tissue remodeling processes.

Cardiac dysfunction, one of the major complications of MS, was notably improved following AAL treatment. HFHFr-fed rats exhibited increases in heart weight, reduction in EF and FS, and prolongation of QRS and QT intervals, all of which indicate systolic dysfunction and electrical instability. AAL administration reversed these abnormalities, indicating improvement in myocardial contractility and electrical conduction. Circulating biomarkers of cardiac injury (CK-MB, CPK, and LDH) were significantly decreased in AAL-treated groups. Histological staining revealed attenuated myocardial fibrosis, and gene expression analysis showed downregulation of TGF-β, Col1, and Col3 expression—key mediators of fibrotic remodeling [47, 48]. In addition, the expression of pro-inflammatory cytokines such as TNF-α and IL-6 was reduced, suggesting that suppression of inflammation may contribute to the improvement in cardiac function.

Renal protective effects were also observed. HFHFr feeding caused characteristic renal alterations such as glomerular basement membrane (GBM) thickening, tubular injury, and interstitial expansion, all of which were alleviated by AAL administration [49–51]. Markers of renal function including plasma creatinine, BUN, and creatinine clearance were improved. Electrolyte imbalances were also corrected, suggesting recovery of osmoregulatory capacity. These effects are consistent with the anti-inflammatory and antioxidant properties reported for atractylodin, a major component of AAL [52, 53]. Taken together, these results indicate that AAL mitigated both structural damage and functional decline in the kidneys.

Vascular dysfunction, which plays a central role in the progression of MS-related organ damage, was also alleviated. HFHFr-fed rats displayed elevated systolic blood pressure and reduced peripheral blood flow, indicating increased vascular resistance and endothelial impairment [46, 54, 55]. AAL treatment normalized blood pressure, enhanced tail blood flow, improved aortic relaxation responses to acetylcholine, and reduced collagen accumulation in the vascular wall, as confirmed by Picrosirius red staining. These vascular improvements may have reduced cardiac afterload and enhanced tissue perfusion, indirectly supporting improvements in heart and kidney function.

At the systemic metabolic level, AAL exerted broad benefits. Fasting blood glucose, insulin, HOMA-IR, and leptin levels were reduced following AAL treatment [8, 56–59]. Physical measures such as body weight, abdominal circumference, and fat pad mass also decreased. In addition, lipid profiles improved, with reductions in triglycerides and total cholesterol and an increase in HDL-C levels. Gene expression analysis revealed that AAL suppressed lipogenesis-related genes such as SREBP-1 and LXR [60], suggesting a mechanism for improved lipid metabolism and reduced fat accumulation.

Olmesartan, an angiotensin II receptor blocker (ARB), was used as a reference drug due to its established roles in reducing blood pressure, insulin resistance, and cardiovascular remodeling [61–64]. While both AAL and olmesartan improved blood pressure and metabolic outcomes, AAL uniquely influenced tissue structure and inflammatory gene expression. This distinction highlights the multi-target actions of botanical agents like AAL, which can modulate diverse physiological systems simultaneously. Therefore, olmesartan was included as a phenotypic comparator to evaluate the therapeutic scope of AAL.

Several limitations should be considered when interpreting the results. First, the study was conducted exclusively in male rats, which limits the ability to generalize the findings across sexes. Since hormonal differences can influence metabolism, future studies including female animals are needed to assess sex-specific responses. Second, although the presence of atractylodin in AAL was confirmed via HPLC, quantitative validation parameters such as calibration curves, detection limits, and reproducibility across extract batches were not established, which restricts the standardization of the preparation [65, 66]. Third, mechanistic analysis at the protein level was not fully conducted. Preliminary Western blotting for AMPK and TGF-β/Smad pathways was attempted but did not yield statistically significant results, likely due to biological variability and limited sample size. Therefore, those data were omitted from the main figures. Validation of key signaling pathways will be essential in future studies. Fourth, the histological analysis was initially descriptive. Quantitative morphometric analysis using ImageJ software has been incorporated to measure parameters such as glomerular area and collagen volume fraction, providing more objective and reproducible histological data. Lastly, translational interpretation should be approached with caution. No pharmacokinetic or toxicological evaluations were performed, and dose conversion for human application was not addressed. This is particularly important due to the presence of bioactive components such as atractylodin, which may exert off-target effects.

## Conclusion

In conclusion, *Atractylodes lancea* rhizome (AAL) exhibited significant therapeutic effects in a rat model of metabolic syndrome by improving glucose and lipid metabolism, reducing insulin resistance, and alleviating dysfunction in the heart, blood vessels, and kidneys. These effects appear to be mediated through anti-inflammatory and anti-fibrotic pathways, along with enhanced endothelial function and suppression of pathological remodeling. Collectively, the findings support the potential utility of AAL as a multi-target approach for managing cardio-renal complications associated with metabolic syndrome.

## Supplementary Information

Supplementary Material 1

Supplementary Material 2

## Data Availability

No datasets were generated or analysed during the current study.

## References

[1] Tanner RM, Brown TM, Muntner P. Epidemiology of obesity, the metabolic syndrome, and chronic kidney disease. Curr Hypertens Rep. 2012;14(2):152–9. 22318504 10.1007/s11906-012-0254-y

[2] Ge L, Sadeghirad B, Ball GDC, da Costa BR, Hitchcock CL, Svendrovski A, Kiflen R, Quadri K, Kwon HY, Karamouzian M, Adams-Webber T, Ahmed W, Damanhoury S, Zeraatkar D, Nikolakopoulou A, Tsuyuki RT, Tian J, Yang K, Guyatt GH, Johnston BC. Comparison of dietary macronutrient patterns of 14 popular named dietary programmes for weight and cardiovascular risk factor reduction in adults: systematic review and network meta-analysis of randomised trials. BMJ. 2020;369:m696. 32238384 10.1136/bmj.m696 PMC7190064

[3] Cameron AJ, Shaw JE, Zimmet PZ. The metabolic syndrome: prevalence in worldwide populations. Endocrinol Metab Clin North Am. 2004;33(2):351–75. 15158523 10.1016/j.ecl.2004.03.005

[4] Blaton V. How is the metabolic syndrome related to the dyslipidemia?? EJIFCC. 2007;18(1):15–22. 29632463 PMC5875077

[5] Antoniolli LP, Nedel BL, Pazinato TC, de Andrade Mesquita L, Gerchman F. Accuracy of insulin resistance indices for metabolic syndrome: a cross-sectional study in adults. Diabetol Metab Syndr. 2018;10(1):65. 30151057 10.1186/s13098-018-0365-y PMC6102896

[6] Lozano L, Werf RV, Bietiger W, Seyfritz E, Peronet C, Pinget M, Jeandidier N, Maillard E, Marchioni E, Sigrist S, Dal S. High-fructose and high-fat diet-induced disorders in rats: impact on diabetes risk, hepatic and vascular complications. Nutr Metab (Lond). 2016;13:15. 26918024 10.1186/s12986-016-0074-1 PMC4766713

[7] Malik S, Wong ND, Franklin SS, Kamath TV, L’Italien GJ, Pio JR, et al. Impact of the metabolic syndrome on mortality from coronary heart disease, cardiovascular disease, and all causes in United States adults. Circulation. 2004;110(10):1245–50. 15326067 10.1161/01.CIR.0000140677.20606.0E

[8] Farag MM, Ashour EH, El-Hadidy WF. Amelioration of high fructose diet-induced insulin resistance, hyperuricemia, and liver oxidative stress by combined use of selective agonists of PPAR-a and PPAR-y in rats. Dubai Med J. 2020;3:76–86.

[9] Wilcox G. Insulin and insulin resistance. Clin Biochem Rev. 2005;26(2):19–39. 16278749 PMC1204764

[10] Grundy SM. Metabolic syndrome: connecting and reconciling cardiovascular and diabetes worlds. J Am Coll Cardiol. 2006;47(6):1093–100. 16545636 10.1016/j.jacc.2005.11.046

[11] Nojima H, Kimura I, Kimura M. Blocking action of succinylcholine with beta-eudesmol on acetylcholine-activated channel activity at endplates of single muscle cells of adult mice. Brain Res. 1992;575(2):337–40. 1571792 10.1016/0006-8993(92)90101-e

[12] Roberts CK, Hevener AL, Barnard RJ. Metabolic syndrome and insulin resistance: underlying causes and modification by exercise training. Compr Physiol. 2013;3:1–58. 23720280 10.1002/cphy.c110062 PMC4129661

[13] Jia G, Hill MA, Sowers JR. Diabetic cardiomyopathy: an update of mechanisms contributing to this clinical entity. Circ Res. 2018;122(4):624–38. 29449364 10.1161/CIRCRESAHA.117.311586 PMC5819359

[14] Landecho MF, Colina I, Huerta A, Fortuño A, Zalba G, Beloqui O. Relación entre Las fases precoces de La enfermedad renal y El síndrome metabólico [Connection between the early phases of kidney disease and the metabolic syndrome]. Rev Esp Cardiol. 2011;64(5):373–8. 21481511 10.1016/j.recesp.2010.11.011

[15] Li Cavoli G, Passantino R, Ferrantelli A, Tralongo A, Servillo F, Li Cavoli TV, Tralongo P, Palmeri M, Ferrantelli G, Ugo R. Acute kidney injury in a patient with metabolic syndrome. Bioimpacts. 2015;5(3):155–7. 26457254 10.15171/bi.2015.13 PMC4597164

[16] Guijarro C, Keane WF. Lipid-induced glomerular injury. Nephron. 1994;67(1):1–6. 8052348 10.1159/000187881

[17] Nagase M, Yoshida S, Shibata S, Nagase T, Gotoda T, Ando K, et al. Enhanced aldosterone signaling in the early nephropathy of rats with metabolic syndrome: possible contribution of fat-derived factors. J Am Soc Nephrol. 2006;17(12):3438–46. 17082236 10.1681/ASN.2006080944

[18] Marassi M, Fadini GP. The cardio-renal-metabolic connection: a review of the evidence. Cardiovasc Diabetol. 2023;22(1):195. Published 2023 Jul 31. 37525273 10.1186/s12933-023-01937-x PMC10391899

[19] Jiang Z, Lu W, Zeng Q, Li D, Ding L, Wu J. High glucose-induced excessive reactive oxygen species promote apoptosis through mitochondrial damage in rat cartilage endplate cells. J Orthop Res. 2018;36(9):2476–83. 29663489 10.1002/jor.24016

[20] González P, Lozano P, Ros G, Solano F. Hyperglycemia and oxidative stress: an integral, updated and critical overview of their metabolic interconnections. Int J Mol Sci. 2023;24(11):9352. Published 2023 May 27. 37298303 10.3390/ijms24119352 PMC10253853

[21] Martín-Carro B, Martín-Vírgala J, Fernández-Villabrille S, et al. Role of Klotho and AGE/RAGE-Wnt/β-Catenin signalling pathway on the development of cardiac and renal fibrosis in diabetes. Int J Mol Sci. 2023;24(6):5241. Published 2023 Mar 9. 36982322 10.3390/ijms24065241 PMC10049403

[22] Leurs P, Lindholm B. The AGE-RAGE pathway and its relation to cardiovascular disease in patients with chronic kidney disease. Arch Med Res. 2013;44(8):601–10. 24231387 10.1016/j.arcmed.2013.11.002

[23] Stanciu SM, Jinga M, Miricescu D, et al. mTOR dysregulation, insulin resistance, and hypertension. Biomedicines. 2024;12(8):1802. Published 2024 Aug 8. 39200267 10.3390/biomedicines12081802 PMC11351979

[24] Aroor AR, Demarco VG, Jia G, et al. The role of tissue renin-angiotensin-aldosterone system in the development of endothelial dysfunction and arterial stiffness. Front Endocrinol (Lausanne). 2013;4:161. 24194732 10.3389/fendo.2013.00161 PMC3810594

[25] Nishi H, Higashihara T, Inagi R. Lipotoxicity in kidney, heart, and skeletal muscle dysfunction. Nutrients. 2019;11(7):1664. Published 2019 Jul 20. 31330812 10.3390/nu11071664 PMC6682887

[26] D’Elia JA, Weinrauch LA. Lipid toxicity in the Cardiovascular-Kidney-Metabolic syndrome (CKMS). Biomedicines. 2024;12(5):978. Published 2024 Apr 29. 38790940 10.3390/biomedicines12050978 PMC11118768

[27] Raabo E, Terkildsen TC. On the enzymatic determination of blood glucose. Scand J Clin Lab Invest. 1960;12:402–7. 13738785 10.3109/00365516009065404

[28] Han HK, Choi EY. Effects of atractylodes lancea on plasma glucose and lipid profile in streptozotocin induced diabetic rats. Korean J Food Nutr. 2020;33(5):544–50.

[29] Kim HJ, Han J, Shin YK, Kang SS. Modulatory effects of atractylone on AMPK signaling in adipocytes. Biochem Biophys Res Commun. 2018;495(1):934–40.

[30] Zhao T, Tang H, Xie L, Zheng Y. Inhibitory effects of sesquiterpenoids from *atractylodes lancea* on inflammatory responses in RAW264.7 cells. Int Immunopharmacol. 2020;83:106475.32283508

[31] Li Y, Chen J, Xiong Y, Wang J, Xu Y. Atractylone suppresses LPS-induced inflammation by inhibiting NF-κB signaling in macrophages. J Ethnopharmacol. 2021;267:113545.33157221

[32] Han LK, Zheng YN, Xu BJ, Okuda H, Kimura Y. Anti-obesity effects of crude drugs from traditional Chinese medicine on mice fed a high-fat diet. Phytother Res. 2005;19(5):360–5.

[33] Yamamoto S, Nagata S, Yamashita Y, Tsuji A, Tani T. Effects of atractylodes lancea extracts on glucose tolerance in obese mice. J Nutr Sci Vitaminol (Tokyo). 2015;61(5):411–7.

[34] Zhou X, Xiao X, Wang Z, Cai J. Hepatoprotective effects of atractylone on non-alcoholic fatty liver disease in mice by attenuating oxidative stress and inflammation. Biomed Pharmacother. 2021;137:111293.33485120

[35] Park KH, Kim HK, Lee SH, Jung DH, Kim JB. Blood pressure-lowering effects of *atractylodes lancea* in spontaneously hypertensive rats. J Korean Med Sci. 2019;34(12):e95.30914906

[36] Cai W, Zhang K, Li P, He H, Tan Y. Sesquiterpenoids from *Atractylodes lancea* improve myocardial function by attenuating oxidative damage. Front Cardiovasc Med. 2020;7:30.32258062

[37] Wang X, Song Y, Zhang Y, Wang S. Cardioprotective effects of atractylone on myocardial ischemia-reperfusion injury via modulation of PI3K/Akt signaling pathway. J Pharmacol Sci. 2022;148(3):233–42.

[38] Wang J, Chen Y, Zhao Z, Shi S. Atractylone alleviates renal fibrosis by regulating TGF-β/Smad signaling in a rat model of renal fibrosis. J Ethnopharmacol. 2020;253:112646.32027997

[39] Liu D, Zhang J, Xie Y, Wu L. Anti-inflammatory and diuretic effects of atractylodes lancea extract in animal models of acute kidney injury. Phytomedicine. 2021;91:153695.

[40] Sun H, Liu Z, Wu M, Guo Q. Protective effects of atractylodes lancea on acute kidney injury by reducing inflammatory cytokines and oxidative stress in rats. BMC Complement Altern Med. 2020;20(1):65.

[41] Wang O, Liu J, Cheng Q, Guo X, Wang Y, Zhao L, Zhou F, Ji B. Effects of ferulic acid and γ-oryzanol on high-fat and high-fructose diet-induced metabolic syndrome in rats. PLoS One. 2015;10(2): e0118135. 25646799 10.1371/journal.pone.0118135 PMC4315454

[42] Zhang J, Wang O, Guo Y, Wang T, Wang S, Li G, et al. Effect of increasing doses of linoleic and α-linolenic acids on high-fructose and high-fat diet induced metabolic syndrome in rats. J Agric Food Chem. 2016;64(4):762–72. 26743332 10.1021/acs.jafc.5b04715

[43] Al-Majed AA, Bakheit AHH, Abdel Aziz HA, Al-Jallal AAM. Olmesartan Profiles Drug Subst Excip Relat Methodol. 2017;42:241–86. 28431778 10.1016/bs.podrm.2017.02.005

[44] Matthews DR, Hosker JP, Rudenski AS, Naylor BA, Treacher DF, Turner RC. Homeostasis model assessment: insulin resistance and beta-cell function from fasting plasma glucose and insulin concentrations in man. Diabetologia. 1985;28(7):412–9. 3899825 10.1007/BF00280883

[45] Kinosian B, Glick H, Garland G. Cholesterol and coronary heart disease: predicting risks by levels and ratios. Ann Intern Med. 1994;121(9):641–7. 7944071 10.7326/0003-4819-121-9-199411010-00002

[46] Eckel RH, Grundy SM, Zimmet PZ. The metabolic syndrome. Lancet. 2005;365(9468):1415–28. 15836891 10.1016/S0140-6736(05)66378-7

[47] Singh D, Rai V, Agrawal DK. Regulation of collagen I and collagen III in tissue injury and regeneration. Cardiol Cardiovasc Med. 2023;7(1):5–16. 36776717 10.26502/fccm.92920302 PMC9912297

[48] Hayden MR, Chowdhury N, Govindarajan G, Karuparthi PR, Habibi J, Sowers JR. Myocardial myocyte remodeling and fibrosis in the cardiometabolic syndrome. J Cardiometab Syndr. 2006;1(5):326–33. 17679785 10.1111/j.1559-4564.2006.05626.x

[49] Odermatt A. The western-style diet: a major risk factor for impaired kidney function and chronic kidney disease. Am J Physiol-Renal Physiol. 2011;301(5):F919-31. 21880837 10.1152/ajprenal.00068.2011

[50] Yustisia I, Tandiari D, Cangara MH, Hamid F, Daud NA. A high-fat, high-fructose diet induced hepatic steatosis, renal lesions, dyslipidemia, and hyperuricemia in non-obese rats. Heliyon. 2022;8(10):e10896. 36247176 10.1016/j.heliyon.2022.e10896 PMC9562237

[51] de Castro UG, dos Santos RA, Silva ME, de Lima WG, Campagnole-Santos MJ, Alzamora AC. Age-dependent effect of high-fructose and high-fat diets on lipid metabolism and lipid accumulation in liver and kidney of rats. Lipids Health Dis. 2013;12:136. 24044579 10.1186/1476-511X-12-136 PMC3849586

[52] Xu L, Zhou Y, Xu J, et al. Anti-inflammatory, antioxidant and anti-virulence roles of atractylodin in attenuating *Listeria monocytogenes* infection. Front Immunol. 2022;13:977051 (**Published 2022 Oct 28**). 36389842 10.3389/fimmu.2022.977051 PMC9651212

[53] Chen L, Tang YL, Liu ZH, Pan Y, Jiao RQ, Kong LD. Atractylodin inhibits fructose-induced human podocyte hypermotility via anti-oxidant to down-regulate TRPC6/p-CaMK4 signaling. Eur J Pharmacol. 2021;913:174616. 34780752 10.1016/j.ejphar.2021.174616

[54] Bunbupha S, Prasarttong P, Poasakate A, Maneesaib P, Pakdeechote P. Imperatorin alleviates metabolic and vascular alterations in high-fat/high-fructose diet-fed rats by modulating adiponectin receptor 1, eNOS, and p47phox expression. Eur J Pharmacol. 2021;899:174010. 33711309 10.1016/j.ejphar.2021.174010

[55] Jin J, Liu J, Luo Y, He H, Zheng X, Zheng C, Huang Y, Chen Y. High fructose induces dysfunctional vasodilatation via PP2A-mediated eNOS Ser1177 dephosphorylation. Nutr Metab (Lond). 2022;19(1): 24. 35331293 10.1186/s12986-022-00659-3 PMC8944156

[56] Kim CH, Kim MS, Youn JY, Park HS, Song HS, Song KH, Park JY, Lee KU. Lipolysis in skeletal muscle is decreased in high-fat-fed rats. Metabolism. 2003;52(12):1586–92. 14669160 10.1016/s0026-0495(03)00328-7

[57] Antuna-Puente B, Disse E, Rabasa-Lhoret R, Laville M, Capeau J, Bastard JP. How can we measure insulin sensitivity/resistance? Diabetes Metab. 2011;37(3):179–88. 21435930 10.1016/j.diabet.2011.01.002

[58] Antunes LC, Elkfury JL, Jornada MN, Foletto KC, Bertoluci MC. Validation of HOMA-IR in a model of insulin-resistance induced by a high-fat diet in Wistar rats. Arch Endocrinol Metab. 2016;60(2):138–42. 27191048 10.1590/2359-3997000000169

[59] Prpic V, Watson PM, Frampton IC, Sabol MA, Jezek GE, Gettys TW. Differential mechanisms and development of leptin resistance in A/J versus C57BL/6J mice during diet-induced obesity. Endocrinology. 2003;144(4):1155–63. 12639896 10.1210/en.2002-220835

[60] Lu H, Lei X, Winkler R, John S, Kumar D, Li W, Alnouti Y. Crosstalk of hepatocyte nuclear factor 4a and glucocorticoid receptor in the regulation of lipid metabolism in mice fed a high-fat-high-sugar diet. Lipids Health Dis. 2022;21(1): 46. 35614477 10.1186/s12944-022-01654-6 PMC9134643

[61] Gardner SF, Franks AM. Olmesartan medoxomil: the seventh angiotensin receptor antagonist. Ann Pharmacother. 2003;37(1):99–105. 12503943 10.1345/aph.1C197

[62] Fliser D, Buchholz K, Haller H, EUropean. Trial on Olmesartan and Pravastatin in inflammation and atherosclerosis (EUTOPIA) investigators. Antiinflammatory effects of angiotensin II subtype 1 receptor blockade in hypertensive patients with microinflammation. Circulation. 2004;110(9):1103–7. 15313950 10.1161/01.CIR.0000140265.21608.8E

[63] Rind L, Mahmood T, Siddiqui MH, et al. From hypertension to beyond: unraveling the diverse mechanisms of Olmesartan in disease modulation. Drug Res (Stuttg). 2024;74(3):93–1016. 38350635 10.1055/a-2244-3136

[64] Yushko K, Koval S, Snihurska I. Olmesartan improves left ventricular hypertrophy in patients with hypertension and type 2 diabetes with low angiotensin-(1–7) blood levels. Eur Heart J. 2020;41(Supplement_2): ehaa946.2775.

[65] Cho HD, Kim U, Suh JH, Eom HY, Kim J, Lee SG, et al. Classification of the medicinal plants of the genus *Atractylodes* using high-performance liquid chromatography with diode array and tandem mass spectrometry detection combined with multivariate statistical analysis. J Sep Sci. 2016;39(7):1286–94. 26888213 10.1002/jssc.201501279

[66] Shrivastava A. Methods for the determination of limit of detection and limit of quantitation of the analytical methods. Chron Young Sci. 2011;2(1):21–5. 10.4103/2229-5186.79345.

