# Supplementary figures and images for: Therapeutic potential of Atractylodes lancea in restoring cardio-renal function in rats with diet-induced metabolic syndrome
Source: BMC Complement Med Ther. 2025 Sep 30;25:338. doi: 10.1186/s12906-025-05074-8 (PMC12482687; doi:10.1186/s12906-025-05074-8)

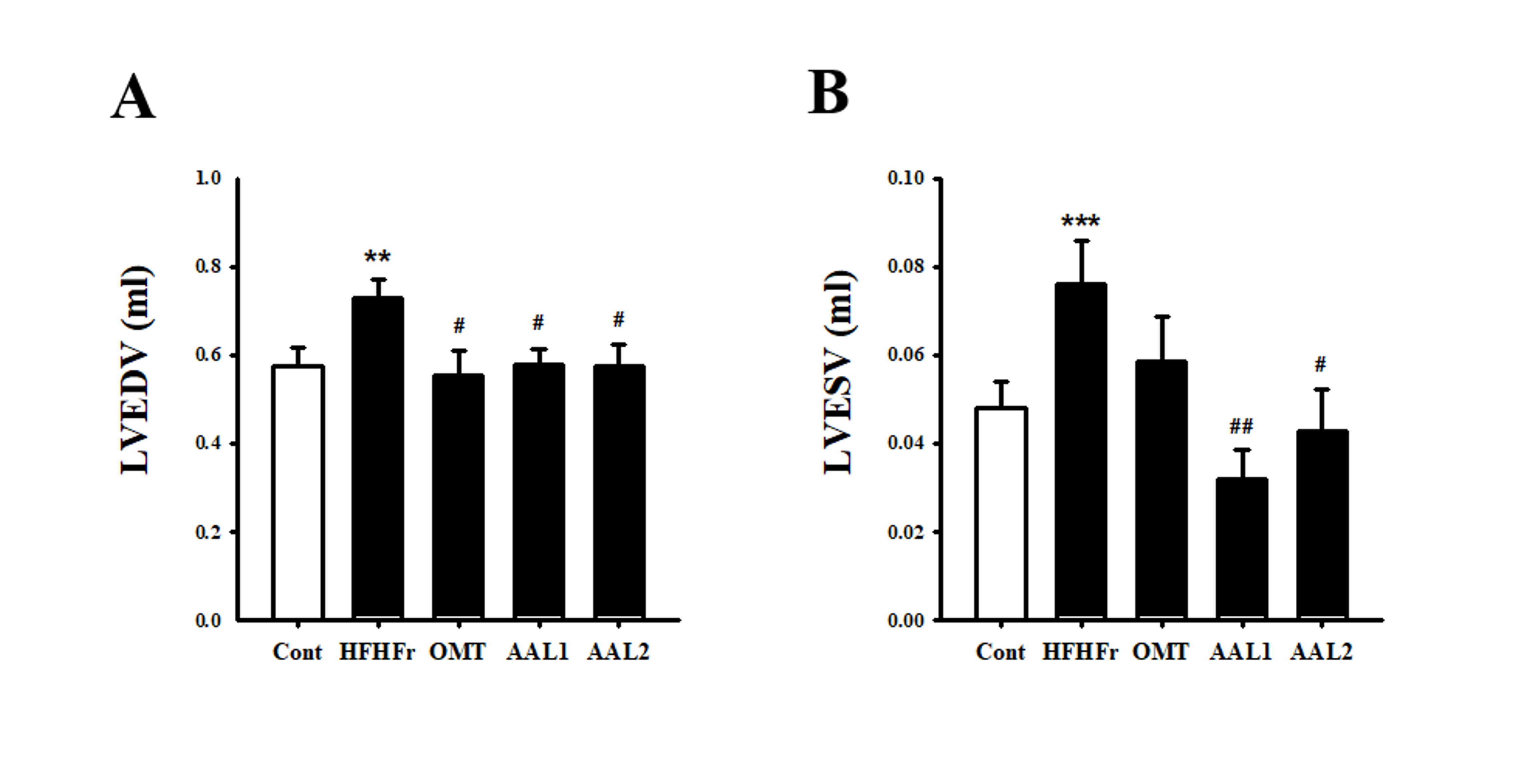

Supplement: Supplementary file 1 — Supplementary Material 1 [file 12906_2025_5074_MOESM1_ESM.jpg]
