# Supplementary material for: Therapeutic potential of Atractylodes lancea in restoring cardio-renal function in rats with diet-induced metabolic syndrome
Source: BMC Complement Med Ther. 2025 Sep 30;25:338. doi: 10.1186/s12906-025-05074-8 (PMC12482687; doi:10.1186/s12906-025-05074-8)
Supplement: Supplementary file 2 — Supplementary Material 2 [file 12906_2025_5074_MOESM2_ESM.pptx]

## Slide 1
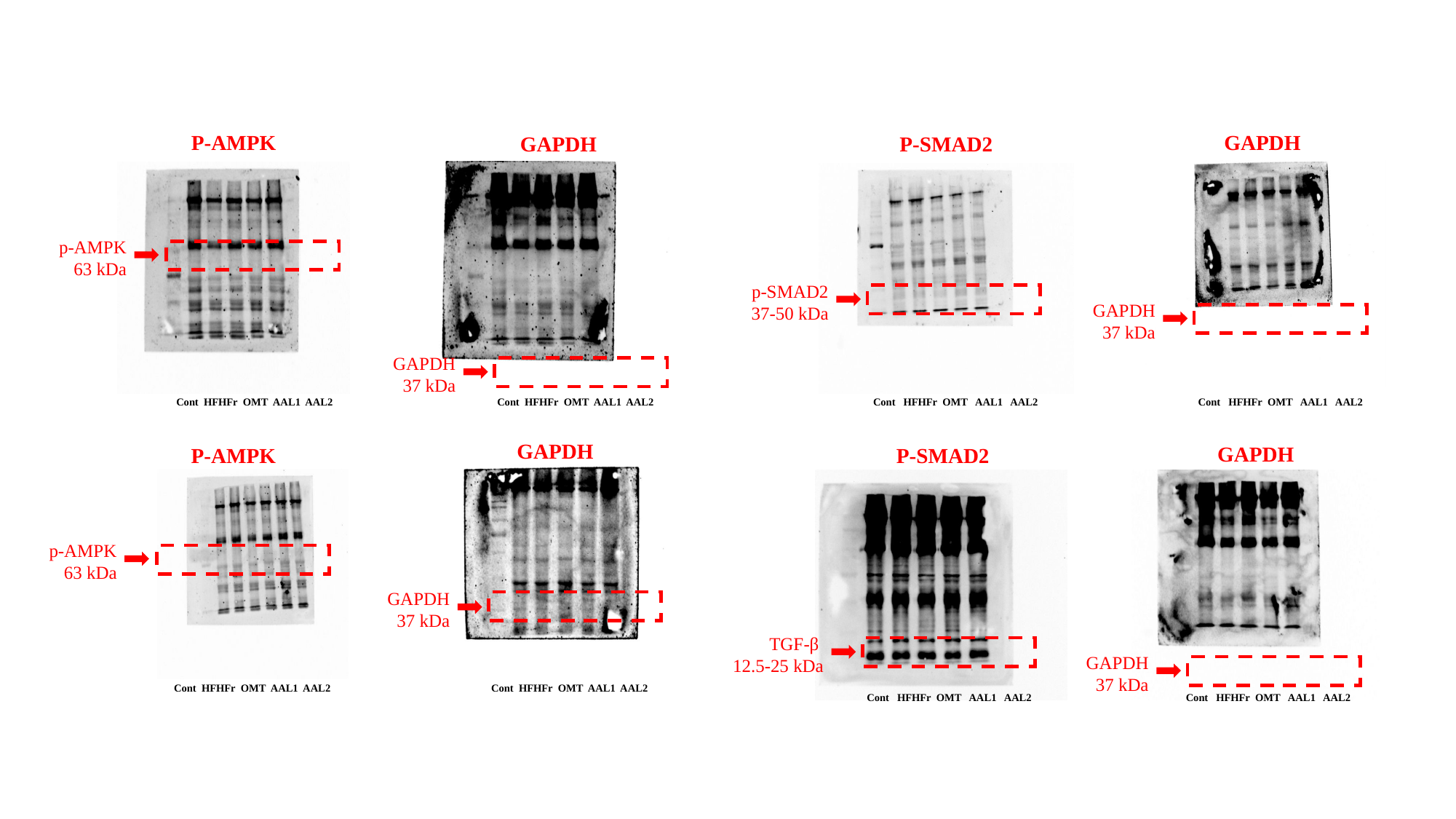

GAPDH
GAPDH
37 kDa
Cont HFHFr OMT AAL1 AAL2
P-AMPK
p-AMPK
 63 kDa
Cont HFHFr OMT AAL1 AAL2
GAPDH
GAPDH
37 kDa
Cont HFHFr OMT AAL1 AAL2
P-SMAD2
p-SMAD2 37-50 kDa
Cont HFHFr OMT AAL1 AAL2
GAPDH
GAPDH
37 kDa
Cont HFHFr OMT AAL1 AAL2
GAPDH
GAPDH
37 kDa
Cont HFHFr OMT AAL1 AAL2
P-AMPK
p-AMPK
 63 kDa
Cont HFHFr OMT AAL1 AAL2
P-SMAD2
TGF-β
12.5-25 kDa
Cont HFHFr OMT AAL1 AAL2
